# Noise, Fake News, and Tenacious Bayesians

**DOI:** 10.3389/fpsyg.2022.797904

**Published:** 2022-05-03

**Authors:** Dorje C. Brody

**Affiliations:** Department of Mathematics, University of Surrey, Guildford, United Kingdom

**Keywords:** noise, signal processing, communication theory, disinformation, electoral competition, marketing, confirmation bias

## Abstract

A modeling framework, based on the theory of signal processing, for characterizing the dynamics of systems driven by the unraveling of information is outlined, and is applied to describe the process of decision making. The model input of this approach is the specification of the flow of information. This enables the representation of (i) reliable information, (ii) noise, and (iii) disinformation, in a unified framework. Because the approach is designed to characterize the dynamics of the behavior of people, it is possible to quantify the impact of information control, including those resulting from the dissemination of disinformation. It is shown that if a decision maker assigns an exceptionally high weight on one of the alternative realities, then under the Bayesian logic their perception hardly changes in time even if evidences presented indicate that this alternative corresponds to a false reality. Thus, confirmation bias need not be incompatible with Bayesian updating. By observing the role played by noise in other areas of natural sciences, where noise is used to excite the system away from false attractors, a new approach to tackle the dark forces of fake news is proposed.

## 1. Introduction

The term “fake news” traditionally is understood to mean false newspaper stories that have been fabricated to enhance the sales of the paper. While unethical, in most cases they are not likely to create long-lasting serious damages to society. However, since the 2016 US presidential election and the 2016 “Brexit” referendum in the UK on the membership of the European Union, this phrase has been quoted more frequently, with the understanding that it refers to deliberate disseminations of false information with an intent to manipulate the public for political or other purposes. The concept of fake news in the latter sense, of course, has been around for perhaps as long as some 3,000 years, and historically it has often been implemented in the context of conflicts between nations or perhaps even between corporations. Hence there is nothing new in itself about fake news, except that the rapid development of the Internet over the past two decades has facilitated its application in major democratic processes in a way that has not been seen before, and this has not only attracted attention of legislators (Collins et al., 2019; Gallo and Cho, 2021) but also generated interests in academic studies of the phenomenon, its implications and prevention (Allcott and Gentzkow, 2017; Shu et al., 2017; Bastos and Mercea, 2018; Bovet and Makse, 2019; Grinberg et al., 2019; Rajabi et al., 2019; Sample et al., 2019; Scheufele and Krause, 2019; Connor Desai et al., 2020; Roozenbeek et al., 2020, to name a few). The purpose of the present paper is to contribute toward this endeavor by applying techniques of communication theory to develop a general framework for characterizing the dynamical behaviors of systems (for example, a group of people) driven by the flow of information, irrespective of whether the information is true or false.

The idea that social science, more generally, can only be properly understood by means of communication theory, for, communication is the building block of any community and hence society, was advocated by Wiener long ago (Wiener, 1954), although its practical implementation has only been developed over the past 20 years in the context of financial applications (Brody et al., 2022). When it comes to the study of the impact of information revelation, whether the information is reliable or not, in particular, the techniques of communication theory become especially effective. This follows from the observation that the change in the behavior of a decision maker that we intend to model results directly from communicating information. Based on this observation, a systematic investigation of the effects of fake news in the context of electoral competition and referendum, from the viewpoint of information transmission, was initiated in Brody and Meier (2022); first appeared in 2018, and developed further in Brody (2019). The concepts underlying these works are elaborated here in greater detail, with the view toward developing measures to counter the negative impacts of deliberate or malicious disinformation that misguide the public.

In more specific terms, to study the impact of disinformation, it is indispensable that information, noise such as rumors and speculations, disinformation, the rate of information revelation, and so on, are all represented by quantities that take numerical values. Otherwise, scientifically meaningful analysis, such as determining the likelihood of certain events to take place, cannot be applied. In probability theory, this idea is represented by the concept of random variables that assign numerical values to outcomes of chance. To study the impact of disinformation, or more generally to study the dynamics of a system governed by information revelation, therefore, the information-providing random time series (which may or may not contain disinformation) will be modeled. Given this “information process” it is then possible to apply powerful and well established techniques of communication theory to study virtually all dynamical properties of the system, including the statistics of future events. In fact, as shown below, the method is sufficiently versatile to the extent that it allows for the numerical simulation of an event that occurs with zero probability—a simulation of what one might call an alternative fact. The fundamental idea underpinning the present approach is that if a decision maker were to follow Bayesian logic (Bayes, 1763) for assessing uncertain events, then the statistics of their behavior can be predicted from a simple mathematical deduction, provided that the flow of information is specified. This motivates us to model the information flow as the starting point so as to *derive* the dynamical behaviors of people driven by information revelation. This is in contrast to more traditional approaches in mathematical modeling whereby one attempts to model the behavior itself from the outset. The latter approach is problematic in the context of information-driven systems under noisy environments, for, the dependence of the output (behavioral dynamics) on the input (information revelation) is often highly nonlinear.

With this in mind the present paper explains how the flow of information can be modeled, and how the unraveling of information under noisy environments affects a decision maker's perception. Then it is shown how the model can be applied to determine the dynamics of an electoral competition, and, in particular, how a deliberate dissemination of disinformation might affect the outcome of a future election. The two fundamental ways in which the information can be manipulated will be discussed. The paper then introduces the concept, to be referred to as the tenacious Bayesian, that explains how people behave in a seemingly irrational manner if they excessively overweight their beliefs on a false reality, even though they are following the rational Bayesian logic. This shows that an element of confirmation bias can be explained within the Bayesian framework, contrary to what is often asserted in the literature. Finally, the paper proposes a new approach to counter the impact of disinformation, by focusing on the role played by noise, and by borrowing ideas from statistical physics of controlling a system that entails many internal conflicts or frustrated configurations. Specifically, it is common for a complex system to be trapped in a locally stable configuration that is globally suboptimal, because the system has to enter into a highly unstable configuration before it can reach an even more stable one. However, by increasing the noise level the system is excited and becomes unstable, thence by slowly reducing the noise level the system has a chance of reaching an even more stable configuration.

## 2. Decision Dynamics From Information Processing

Decision making arises when one is not 100% certain about the “right” choice, due to insufficient information. The current knowledge relevant to decision making then reflects the *prior* uncertainty. If additional partial information about the quantity of interest arrives, then this *prior* is updated to a *posterior* uncertainty. To see how this transformation works it suffices to consider a simple example of a binary decision—a decision between two alternatives labeled by 0 and 1—under uncertainty. Suppose that we let *X* be the random variable representing a binary decision so that *X* takes the value 0 with probability *p* and *X* equals 1 with probability 1−*p*, where the probabilities reflect the degree of uncertainty. In the context of an electoral competition, one can think of a two-candidate scenario whereby *X* = 0 corresponds to candidate *A* and *X* = 1 corresponds to candidate *B*. Then the probabilities (*p*, 1−*p*) reflect the *a priori* view of a given decision maker—a voter for example. In particular, if *p*>0.5, then candidate *A* is currently preferred over candidate *B*.

With this setup, the decision maker receives additional noisy information about the “correct” value of *X*. For example, one might read an article that conveys the information that voting for candidate *A* is likely to be the correct decision. The idea then is to translate this information into a numerical value so as to be able to understand and characterize how the view of the decision maker, represented by the probabilities (*p*, 1−*p*), is affected by acquiring further information. To model this mathematically, let ϵ denote the random variable representing noise, which is assumed statistically independent of *X*. The origin of noise may be a genuine mistake, or a plain speculation, on the part of the author of the article, or perhaps a simple misinterpretation of the article on the part of the decision maker. The idea thus is to regard the unknown quantity of interest, the value of *X* in this case, as a signal to be inferred, which is obscured by noise. Hence the receiving of noisy information amounts to observing the value of


ξ=X+ϵ.

Because there are two unknowns, *X* and ϵ, and one known, the value of ξ, it is not possible to determine the value of *X*, which reveals the correct choice of action, from this information. Nevertheless, the knowledge of the value of ξ will allow the decision maker to reduce the uncertainty about *X*. As an example, suppose that there is a wide range of rumors and speculations about the value of *X*. If there are many such contributions to the noise, then the law of large numbers implies that it is reasonable to assume ϵ be normally distributed, say, with mean zero and some standard deviation ν. Of course, the nature of noise may not be of Gaussian type, and likewise the signal and noise decomposition in general need not be additive. One of the advantages of the present approach is that depending on the context, it is possible to choose the structure of the information-baring random variable ξ, and proceed to analyse its consequences (see e.g., Brody et al., 2013). However, for illustrative purposes here we shall proceed with the additive Gaussian noise model.

Suppose that the value of ν is relatively small, say, ν = 0.2. This means that the distribution of ϵ is narrowly peaked at ϵ = 0. Suppose further that the value of the observation is ξ = 0.73. In this case there are two possibilities: we have either (*X*, ϵ) = (0, 0.73) or (*X*, ϵ) = (1, −0.27). However, given that the distribution of ϵ is narrowly peaked at ϵ = 0, the realization ϵ = 0.73 is significantly less likely as compared to the event that ϵ = −0.27. Hence after the observation that ξ = 0.73 the prior probabilities (*p*, 1−*p*) will be updated to the posterior probabilities (*p*′, 1−*p*′) in such a way that *p*′ < *p*, whenever ξ>0.5. The exact value of *p*′ will be dependent on the value of *p*, and can be calculated using the Bayes formula:


$$p^{\prime }=\frac{p\rho \left(\xi |X=0\right)}{p\rho \left(\xi |X=0\right)+\left(1-p\right)\rho \left(\xi |X=1\right)},$$

where ρ(ξ|*X* = 0) is the density function of the random variable ξ given the event that *X* = 0, and similarly for ρ(ξ|*X* = 1). Because conditional on the value of *X* the random variable ξ is normally distributed with mean *X* and variance ν^2^, in the present context the Bayes formula gives


$$p^{\prime }=\frac{p}{p+\left(1-p\right)\exp\left(\frac{1}{\nu^{2}}\left(\xi -\frac{1}{2}\right)\right)}.$$

Thus, for instance, if the *a priori* probability is 50–50 so that *p* = 0.5 then we find in this example with ν = 0.2 and ξ = 0.73 that *p*′≈0.0032. In other words, although the value of *X* remains unknown, we can be almost (99.68%) certain that the decision corresponding to *X* = 1 is the correct choice, based on the observation of information relevant to decision making. The example here is consistent with our intuition, owing to the fact that our brains appear capable of subconsciously implementing the Bayes formula at an intuitive level, when it comes to processing signals under noisy environments—for example, in attempting to catch a ball in the air, instead of consciously solving Newton's equations, our brain subconsciously processes the visual signal to achieve the task. (Whether a human brain is capable of subconsciously implementing Bayesian rules in broader contexts might be questionable—one recent proposal (Sanborn and Chater, 2016) is that the brain functions instead as a Bayesian sampler.) Therefore, once the observation is made, our views will be shifted, resulting in actions, such as making a decision. In other words, it is the processing of noisy information that results in the dynamics of decision makers: new information arrives, positions reassessed, and actions taken.

The approach taken here to model the dynamics of decision making is based on the standard formalism of communication theory (Wiener, 1948; Shannon and Weaver, 1949). In communication theory, the random variable *X* represents the signal that has been transmitted, which is obscured by noise, represented here by the random variable ϵ. The task then is to determine the best estimate of *X* given the observation ξ. Because the processing of imperfect information is intrinsic to any decision making under uncertainty, communication theory is highly effective in characterizing dynamical behaviors of people driven by information revelation. Indeed, communication theory has been applied extensively to model dynamical behaviors of financial markets, or more precisely the dynamics of asset prices, over the past two decades (Brody et al., 2022). In the context of a financial market, asset prices change in accordance with transaction decisions. When a market participant makes a decision on an investment, their primary concern is the unknown future return resulting from that investment. By letting *X* be the random variable representing the return of a given investment over a period, whose value is obscured by market noise, it is then possible to arrive at a plausible model for the price dynamics using the techniques of signal processing in communication theory, because the model merely replicates, albeit with some simplifying approximations, what actually takes place in real world—prices change in accordance with the flow of information.

Traditional communication theorists have shied away from applying techniques of signal detection to model behavioral dynamics, for, the random variable *X* appearing in the context of decision making is typically not “transmitted” by the sender of a communication channel. Instead, it represents the quantity of interest that one wishes to infer under uncertainty. In a financial context, for instance, *X* may represent the future return over an investment period, whose value is not known to anyone, so clearly no one is able to transmit the value of *X*. Yet, *X* certainly exists, whose value will be revealed at the end of that investment period. In this case, it is the market as a whole that acts like an abstract communication channel. Likewise, situations are similar for many other decision makings under uncertainties, but it requires a leap of imagination to realize that communication theory provides a unified framework for characterizing the dynamics of information-driven systems even when there is no explicit mechanical device to form a communication channel.

There is another reason why, in spite of its effectiveness, signal processing had not been widely applied to modeling behavioral dynamics, and this has to do with the meaning of random variables in probability. Take, for instance, the case of coin tossing. If the coin is fair, then the outcome head is as likely seen as the outcome tail. But what would be the average? There is no way of answering this question using common language—for sure the coin does not have a “Cecrops” face that is half head and half tail. To make any statistical consideration such as taking the average, it is necessary to assign numerical values to outcomes of chance, called a random variable, so for instance we can associate the number 1 to the outcome head, and 0 to the outcome tail. We can then meaningfully say that the average of the outcome of a fair coin is 0.5 without any difficulty. In a similar vein, to model decision making under uncertainty it is necessary to introduce a random variable to represent different options, and likewise another random variable to represent noise. The idea of assigning numerical values to rumors, speculations, estimations, news bulletins, etc., may appear rather abstract, and it requires another leap in imagination to realize that this is in fact no more abstract than associating the values 0 and 1 to the outcomes of a coin tossing. Indeed, the variable *X* in a decision making refers to the correct choice. The information ξ = *X*+ϵ thus does not refer to observing one's own decision process. Rather, ξ embodies the observation of external information sources in relation to arriving at the correct choice in the decision making.

The example above in which the observation is characterized by the relation ξ = *X*+ϵ is, of course, meant to represent the simplest situation, whereas in real-life decision makings, the noise typically changes in time and is thus represented by a time series {ϵ_*t*_}, where *t* denotes the time variable. In some cases, the nature of available decision options itself may change in time, in which case *X* will also be replaced by a time series. At any rate, in almost all realistic circumstances, the information-providing observation is not a fixed random variable, but rather is given by a time series {ξ_*t*_}. Fortunately, the theory of signal detection and communication is highly developed (Davis, 1977; Liptser and Shiryaev, 2001) so as to allow for a good level of tractability to model many realistic circumstances in decision making.

## 3. Modeling Electoral Competition With Information

An information-based approach to modeling the dynamics of electoral competitions has been introduced recently in Brody and Meier (2022) and in Brody (2019). The idea can be sketched as follows. In the context of an electoral competition, a voter typically has a handful of issues of concern (such as taxation policy, climate policy, education policy, policies on abortion right and gun control, or perhaps the personality of the leader of a political party, &c.), and likewise possesses partial information about how different candidates, if elected, will implement policies to address these issues. Each such issue is then modeled by a random variable so as to assign numerical values to policy positions, and these random variables, whose values represent different policy positions different candidates would implement, play the role of signals whose values the voters wish to identify. Hence for example in the case of a binary issue (for or against), one can assign, say, the values +1 and −1 to the two positions. Each such random variable is referred to as a “factor” and for each electoral factor there is a noisy observation characterized by a time series. Thus voters can only infer the best estimates for the values of these factors, based on available information.

For a given voter, their preferences on different policy positions are then modeled by weights {*w*_*k*_}, which are not necessarily positive numbers. The signs of the weights reflect their preferences on the various issues, while the magnitude |*w*_*k*_| represents the significance of the policy position about the *k*-th issue for that voter. In Brody and Meier (2022) a linear scoring rule was assumed to associate for each candidate a score from a given voter, determined by the weighted average of their best estimates for different factors. That is, the score *S*_*l*_ assigned to candidate *l* by a voter with preference pattern {*w*_*k*_} is given by


$$S_{l}=\underset{k}{\sum }w_{k}E\left[X_{k}\right],$$

where *E*[−] denotes expectation operation. A given voter will then choose to vote for the candidate with the highest score. The importance of imperfect information about the policy positions of the candidates in electoral competitions has been noted before (see e.g., Harrington, 1982; McKelvey and Ordeshook, 1985; Feddersen and Pesendorfer, 1997; Fowler and Margolis, 2014). The approach of Brody and Meier (2022) is to take this idea a step further by modeling the noisy flow of information concerning the values of the policy positions in the form of a time series {ξ_*t*_}, from which the dynamics of the opinion poll statistics can be deduced. This is because the expectation *E*[−] is now replaced by a conditional expectation subject to the noisy information flow regarding the policy positions of the candidates.

Another advantages of this approach, apart from being able to simulate the time development of the conditional expectations of the electoral factors {*X*_*k*_}, is that given the information about the distribution of voter preferences within a group of the population, it is computationally straightforward to sample a large number of voter profiles (the weights {*w*_*k*_}) without going through the costly and time-consuming sampling of the actual voters. Thus, for example, if there were one million voters, and if we have the knowledge of the distribution of voter preferences on different issues, then by sampling from this distribution a million times we can artificially create voter preference patterns, from which we are able to simulate the dynamics of the opinion poll statistics and study their implications. As a consequence, the information-based approach makes large-scale simulation studies and scenario analysis on behavioral pattern feasible, when it comes to systems driven by information revelation under uncertainties.

It should be evident that because the starting points of the formalism based on communication theory are (a) to identify relevant issues and associate to them random variables, called factors, and (b) to build a model for the flow of information for each of the factors, it readily suggests a way to explore how adjustments of information flow (for example, when to release certain information) will affect the statistics of the future (such as the probability of a given candidate winning on the election day). Furthermore, it also suggests a way to model deliberate disinformation and study their impacts. These ideas will be explained in more detail below.

## 4. Disinformation and Their Impacts

The intention of deliberate disinformation—the so-called “fake news”—is, as many people interpret the phrase nowadays, to create a bias in people's minds so as to impact their behaviors and decision makings. But clearly such disinformation will have little impact if the person who receives the information is aware of this. That is, if the person has an advanced knowledge of the facts, then they will not be impacted by false information—although there are suggestions that there can be such “anchoring” effect even among well-informed individuals (Wilson et al., 1996). (The situation is different if a false information is given first, and the truth is revealed subsequently, because in this case the prior belief has been shifted before the facts are revealed.) Because disinformation is not part of the “signal” that in some sense represents truth, it can be viewed as a kind of noise. In the context of a traditional communication channel, on the other hand, while noise is a nuisance, it does not have an intent. In other words, noise does not have an unknown bias. Putting these together, it should be apparent that the release of deliberate disinformation is equivalent to the introduction of a bias into the noise that is undetected by the receiver of the information. Thus, in the case of the earlier example, the observation under disinformation takes the form


η=X+(ϵ+f),

where *f* represents the biased disinformation so that the expectation of *f* is nonzero. If a person receives the value of η, but is unaware of the existence of the term *f* and presumes that it is the value of ξ = *X*+ϵ, then the resulting inference will be misguided. For instance, a positive *f* will misguide people in thinking that the value of *X* is larger than what it actually is, and conversely for a negative *f* people will be misled to the conclusion that the value of *X* is smaller than what it actually is. Again, once one recognizes the need for the introduction of a random variable *f* for representing disinformation so as to allow for a meaningful statistical treatment, it becomes apparent how to model and study behavioral dynamics in the presence of fake news.

Continuing on with this simple example, where *X* is a binary random variable with *a priori* probabilities (*p*, 1−*p*) = (0.5, 0.5) and ϵ is a zero-mean normal random variable with standard deviation ν = 0.2, suppose that disinformation is released so as to enhance the probability that the choice corresponding to *X* = 0 is selected by a decision maker. The decision maker is under the assumption that the observation is of the form ξ = *X*+ϵ. This means, in particular, that the smaller the value of ξ is, the higher the *a posteriori* probability of *X* = 0 is. To enhance the *a posteriori* probability, suppose, in the previous scenario whereby ξ = 0.73, that the released disinformation amounts to the realization that *f* = −0.093. Then the perceived, or deceived *a posteriori* probability is *p*′≈0.032, even though in reality the number ought to be *p*′≈0.0032.

In the above example, the disinformation-induced perceived *a posteriori* probability, although has been enhanced by a factor of ten, remains too small to be of significance in affecting decision making. However, it has to be recognized that in reality the information flow is typically continuous in time, i.e., for real-world applications to modeling behavioral dynamics of the public one has to be working with a time series rather than a single-shot information model considered here. What this means is that while each disinformation may only shift the public perception by a small amount, the impact of a relentless release of disinformation accumulates in time to become significant.

**Figure 1:**
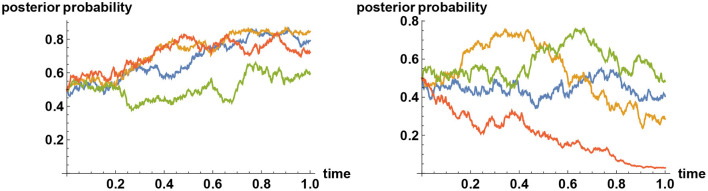
Impact of fake news. The two alternatives are represented by the values *X* = 0 and *X* = 1, but at time zero the uncertainty is at its maximum (the opinion is equally split between the two alternatives). Then noisy observation begins, and the prior opinion is updated in time (expressed in years). Plotted here are sample paths for posterior probabilities that *X* = 1. In all simulations the correct choice is (secretly) preselected to be the one corresponding to *X* = 1. Depending on how the Brownian noise develops the best inference develops differently, as shown here on the left panel, but by waiting sufficiently long, ultimately enough truth is learned and all decisions on the left panel will converge to the correct one, typically by time *t*≈10. The impact of disinformation, released at a random point in time (at *t* = 0.6 here), is shown on the right panel. The disinformation is released at a constant rate in time, intended to mislead the decision maker to select the choice corresponding to *X* = 0. The rate of release is taken to be sufficiently strong that if decision makers are unaware of the disinformation then they will be led to making the incorrect decision (*X* = 0), even though the simulator had preselected the correct decision to be that corresponding to *X* = 1.

To visualize the effect, consider a time-series version of the model in which the time-series {ϵ_*t*_} for noise is represented by a Brownian motion (hence for each increment of time the noise is normally distributed with mean zero and variance equal to that time increment), but the signal *X* remains a zero-one binary random variable, whose value is revealed at a unit rate in time. Thus, the observed time series takes the form


$$\xi_{t}=Xt+\epsilon_{t}$$

in the absence of disinformation, whereas the Brownian noise {ϵ_*t*_} acquires a drift term *f*(*t*−τ) at some point τ in time in the presence of disinformation. Example sample paths with and without a release of fake news are compared in Figure 1.

One of the advantages of the present approach is that a simulator can preselect what is ultimately the ‘correct' decision. Looking at each realization one cannot tell, without waiting for a sufficiently long time, which way the correct decision is going to be. Nevertheless, the simulator is able to select the correct decision in advance and let the simulation run. In this way, a meaningful scenario analysis can be pursued. With this in mind, in Figure 1 sample paths are shown, all of which corresponds to the realization that the decision corresponding to the value *X* = 1 is ultimately the correct decision. On the left panel, starting with a 50–50 prior opinion, the development of the posterior opinion based on the observation of the time series {ξ_*t*_} is shown for four different realizations of the noise {ϵ_*t*_}. Depending on how the noise develops, the realizations will be different, but in all cases, ultimately, by waiting longer than the timescale shown here, the correct decision (selected by the simulator) will be selected by the decision makers. In contrast, if sufficiently strong disinformation intended to guide decision makers toward the incorrect choice (we know that it is incorrect because the simulator did not choose that decision) is released at some point in time, and if nothing is done about this so that decision makers are unaware of this, then ultimately all decisions will converge to the incorrect choice, as shown on the right panel.

It is worth remarking in this connection that in real-world applications there are two situations that arise: One in which the correct decision will be revealed at some point, and one in which this is never revealed. For instance, if the decision is whether to invest in asset *A* or asset *B* over an investment period, then at the end of the period one would discover which investment resulted in a higher yield. For other decisions, for example in the context of an election or referendum, it is often the case that voters will never find out, beyond an informed guess in some cases, which candidate would have been better, because the lost candidates do not have the opportunities to demonstrate the outcomes of their policy implementations. In fact, even for the winning candidate, the merits of their policies may not become apparent, especially when there are long-term consequences. These latter cases amount to having a signal that drops out at some point in time (e.g., on the election day), and hence afterwards the public no longer receives information to improve their assessments. Communication theory allows for the flexibility to handle all these different situations that might arise in reality. For example, if the correct choice is revealed for sure after a finite time horizon, then one can let the noise {ϵ_*t*_} be modeled by a Brownian bridge process that goes to zero at the end of the period (Brody et al., 2022). Alternatively, if the correct choice is never revealed, then one can let the information revelation rate σ introduced below to vanish at some point in time.

## 5. Information control

Besides the impact of disinformation, there is another important ingredient that has to be brought into the analysis when considering the controlling of public behavior. This concerns, for instance, a situation in which there are individuals who are aware of the value of *X* that the public at large are trying to infer. In such a situation, what one might call the information-flow rate, or the signal-to-noise ratio, may be adjusted. To understand this, let us return to our single-shot information model, but this time we have


ξ=σX+ϵ,

where the parameter σ determines the magnitude of the signal. To understand the effect of σ, let us take an extreme case where σ = 100 while *X* is a zero-one binary variable and ϵ is a zero-mean normal variable with a small standard deviation. Then for a given value of the noise ϵ there are two possible observations: ξ = ϵ, or ξ = 100+ϵ. Because the realized value of ϵ will almost certainly be close to zero, we know already that ξ≈0 if *X* = 0 and ξ≈100 if *X* = 1. Hence the effect of σ is to amplify the signal, making it easier to infer the value of *X*. Conversely, suppose that σ = 0.01 in this example. Then we know that ξ≈0 irrespective of whether *X* = 0 or *X* = 1. Hence the observation will be of little help in inferring the value of *X*: the signal is dimmed by having a small value of σ.

With this example in mind it should be evident that the general information model can take the form


η=σX+(ϵ+f).

To control the behavior of the public, one can either introduce the term *f* with a nonzero mean in such a way that the public is unaware of its existence, and hence confuses the contribution of *f* as arising from *X*, or increase (decrease) the value of σ so that the public can arrive at a more reliable inference faster (slower). These are the two fundamental ways in which the public behavior under uncertain environments can be manipulated via information.

It is worth remarking here, incidentally, that if *f* has no bias, then its effect is equivalent to reducing the value of σ, because in this case one is merely enhancing the magnitude of noise. Hence the introduction of purely random disinformation has the effect of slowing down the public from discovering the truth. This may be intuitively apparent, but here it follows as a direct consequence of communication theory. In particular, in the context of an observation involving a more general time series, the timescale of arriving at a reasonable inference about the value of *X* is typically proportional to σ^−2^. This is the timescale for which the amount of uncertainty as measured by the variance of *X* is reduced by more than 50% of the initial uncertainty. Hence if the magnitude of noise is doubled, then it takes four times longer to arrive at the same level of inference.

With the above characterization of the two fundamental ways in which information can be manipulated, it is possible to ask which strategy maximizes the chance of achieving a certain objective, and techniques of communication theory can be used to arrive at both qualitative and quantitative answers. As an example, consider an electoral competition, or a referendum. To simplify the discussion let us assume that the choice at hand is binary, and the information providing process is a time series, where both the noise {ϵ_*t*_} and the information revelation rate {σ_*t*_} are changing in time. If an agent is willing to engage in a strategy to divert the public to a particular outcome based on disinformation, then the example illustrated in Figure 1 shows that it suffices to release “fake news” whose magnitude |*f*_*t*_| is greater than that of the information revelation rate |σ_*t*_|. However, there are two issues for the fake-news advocators: First, the strategy is effective only if the public is unaware of the existence of disinformation. Some people are knowledgable, while others may look it up or consult fact-checking sites. From these, some can infer the probability distribution of disinformation, even though they may not be able to determine the truth of any specific information, and the knowledge of this distribution can provide a sufficient deterrence against the impact of disinformation (Brody and Meier, 2022). Second, a frequent release of information can be costly. For a state-sponsored disinformation unit this may not be an issue, but for most others, it is typically rather costly to disseminate any information to the wider public—for example, by paying a lot of money to the so-called “influencers” to discourage people from being vaccinated against a potentially deadly virus.

From the viewpoint of a fake-news advocator, the cost issue can be addressed by means of signal-processing techniques outlined here. For instance, suppose that for cost reasons there is only one chance of releasing disinformation, whose strength grows initially but over time is damped down, perhaps owing to people discovering the authenticity of the information. In such a scenario one would be interested in finding out the best possible timing to release disinformation so as to maximize, for instance, the probability of a given candidate winning a future election. The answer to such a question of optimisation can be obtained within the present approach (Brody, 2019).

**Figure 2:**
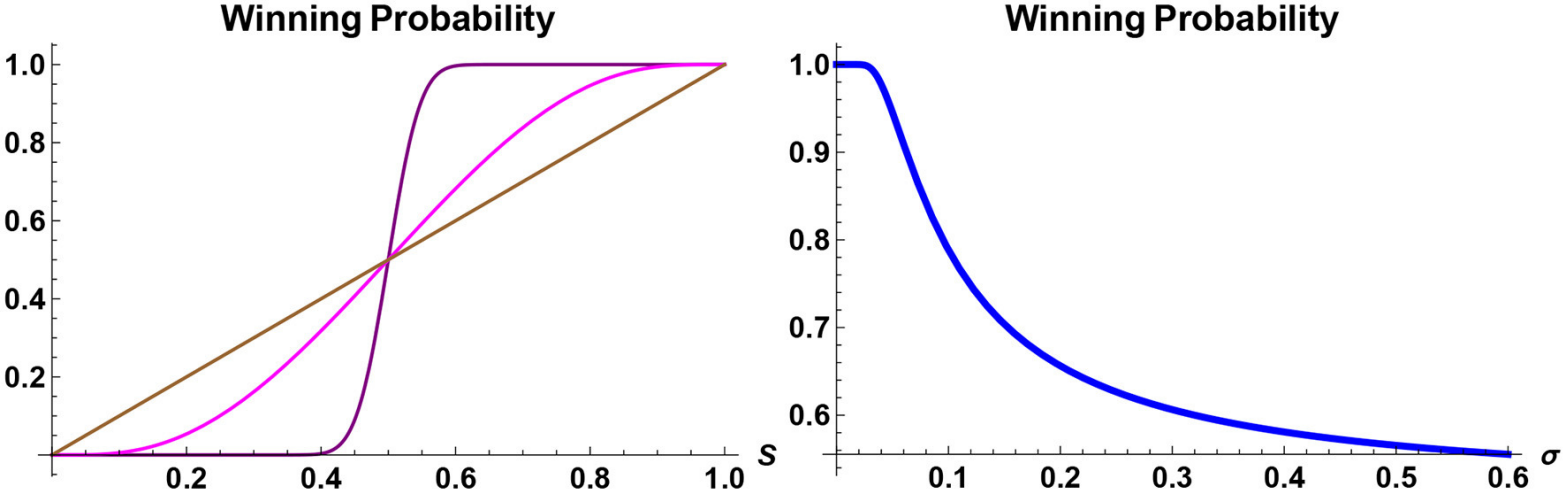
Probability of winning a future election. The winning probability of a candidate in a two-candidate electoral competition, to take place in 1 year time, is plotted. On the left panel, the probabilities are shown as a function of today's support rating *S* for two different values of the information-flow rate: σ = 0.15 (purple) and σ = 0.95 (magenta). If today's poll *S* were an indicator for the winning probability, then it would be given by a straight line (brown), but in reality the probability of winning a future election of a candidate, whose current support rate is *S*>50%, is always greater than *S*. On the right panel, the winning probability is shown as a function of the information-flow rate σ of a candidate whose support rate today is *S* = 52%. If the candidate is leading the poll, then the best strategy is to reveal as little information relevant to the election as possible.

From the viewpoint of an individual, or perhaps a government, who wishes to counter the impact of disinformation, on the other hand, the analysis presented here will allow for the identification of optimal strategies potentially adopted by fake-news advocators so as to anticipate future scenarios and to be prepared. It also provides a way for developing case studies and impact analysis. This is of importance for two reasons. First, the conventional approach to counter the impacts of fake news, namely, the fact checking, although is an indispensable tool, does not offer any insight into the degree of impact caused by fake news. Second, while information-based approach tends to yield results that are consistent with our intuitions, some conclusions that can be inferred from the approach are evident with hindsight but otherwise appear at first to be counterintuitive. Take, for instance, the probability of a given candidate winning a future election, in a two-candidate race, say, candidates *A* and *B*. It can be shown (Brody, 2019) that if the current poll suggests that candidate *A* has *S%* support, then the actual probability of candidate *A* winning the future election is always greater than *S* if *S*>50, and is always less than *S* if *S* < 50. Hence, contrary to a naive intuition, the current poll statistics are not the correct indicators for the actual realized probabilities of election outcomes. Further, the smaller the information flow rate σ is, the greater is the gap between the current poll and the realized winning probability. Thus, for instance, if *S* = 51, say, 6 months before the election day, and if the value of σ is very small, then the projected probability of candidate *A* winning the election is significantly higher than 51%, and in the limit σ tending to zero, it approaches 100% (see Figure 2).

This may at first seem counterintuitive, but with reflection one can understand why this has to be the case. If the value of σ is close to zero, then what this means is that virtually no information about the factor *X* will be revealed between now and the election day 6 months later (here it is assumed for simplicity of discussion that there is only one factor). But without reliable information people do not change their mind spontaneously. Hence if candidate *A* has 51% support today, then without further information 51% of voters will continue to support candidate *A* 6 months later, meaning that the actual probability of winning is closer to 100%. It follows that if a candidate is leading the poll, then it is in their best interest not to reveal any information about their policies or personality, unless there are good reasons in doing so to further enhance the current lead. Conversely, if a candidate is lagging behind the poll then it is in their best interest to reveal as much information as possible, so as to create movements that may change the balance of the poll.

This example naturally lends itself to the second way in which information can be controlled. Namely, to adjust the value of σ. This is a different approach from the one based on releasing disinformation to guide people away from discovering facts. For example, if there is a fact, such as tax return, that a candidate does not wish the public to find out, or if a candidate is leading the poll statistics even though the candidate has no clue about future policies, then the value of σ can be reduced either by not revealing any information or simply by putting out a lot of random noise peripheral to the issue. Alternatively, if the value of *X* is known to a small number of individuals (e.g., the candidates themselves) when it is advantageous for the candidate that the public should discover this, then they are in the position to release more information to enhance the value of σ. In a more general situation where {σ_*t*_} is time dependent, it is possible to design how the information revelation rate should be adjusted in time (Brody, 2019) so as to maximize the objective (for example maximizing the probability of winning an election, or maximizing the sales in the context of advertisements).

## 6. Information Clusters and Tenacious Bayesians

One of the key issues associated with the deliberate dissemination of disinformation in a coordinated and organized manner (for example, by a state-sponsored unit) concerns the fact that although there is a very wide range of information sources readily available, people have the tendency of gathering information from a limited set of sources, resulting in the creation of clusters of people digesting similar information, and this can be exploited by a malicious fake-news advocator. To understand the formation of such clusters, consider the following scenario in a different context. Imagine that there is a wide open space, with a large number of people standing at random, and that these people are instructed to lie down in such a way that they lie as parallel as possible with their neighbors. Or alternatively, the instruction may be that everyone should lie with their heads pointing either north or south, such that they should lie in the same orientation as their neighbors. In theory, there are alignments such that all the people lie in a perfectly parallel configuration (for instance, they all lie with their heads pointing north), but such a configuration will not be realized in reality. The reason is because the instruction that they should lie as parallel as possible with their neighbors is a local one, and a local optimization does not yield a global optimization when there is a wide-ranging complex landscape of possible configurations. As a consequence, what will happen is the formation of vortices or clusters, in the latter case separated by domain walls separating alignment mismatch, where within a cluster people are closely aligned.

Formation of informational clusters are perhaps not dissimilar to this. The highly developed nature of Internet might give the impression that everything is “global” in this information society, but this is not the case because the concept of a neighbor in an information cluster, where people within a cluster digest similar information sources, need not have any relation to a geographical neighbor: a person living across the Atlantic can be a neighbor in the information cluster, while the next door occupant can be from another universe for that matter. As a consequence of the cluster formation, the type of information digested in one cluster tend to differ from that in another cluster. For instance, a regular reader of a left-leaning news paper is unlikely to pick up a right-leaning paper, and *vice versa*—the heights of the domain walls are made higher by the fluidity of Internet, and, in particular, by fake news.

Of course, those belonging to a given cluster are often well aware of the existence of other opinions shared by those in other clusters. Yet, those counter opinions—the so-called “alternative facts”—have seemingly little impact in altering people's opinions, at least in the short term. The reason behind this can be explained from a property of Bayesian logic. Indeed, one of the consequences of the clustering effect is the tendency of placing heavier prior probabilities on positions that are shared by those within the cluster. The phenomenon of overweighting the prior is sometimes referred to as “conservatism” in the literature (El-Gamal and Grether, 1995), although this terminology can be confusing in a political discussion. At any rate, when the prior probability is highly concentrated toward one of the alternatives, and if this happens to be ultimately the “incorrect” choice, then even if counter facts are presented time and again, the prior weight need not change very much for a long time under the Bayesian inference. This phenomenon will be referred to as the “tenacious Bayesian inference” here.

**Figure 3:**
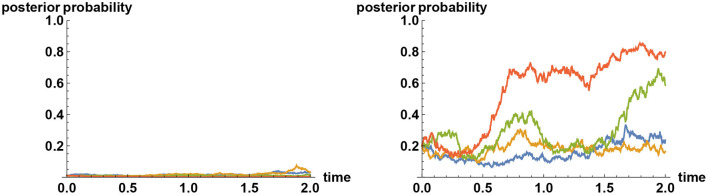
Tenacious Bayesian behavior in a binary decision making. The two alternatives are represented by the values *X* = 0 and *X* = 1, and in all simulations, the “correct” decision is preselected to correspond to the choice *X* = 1. On the left panel, four sample paths for the a posteriori probability that *X* = 1 are shown when the *a priori* probability for the incorrect decision *X* = 0 is given by 99%. Although the evidences presented by the observed time series consistently indicate that *X* = 0 is not the correct choice, the decision makers in these simulations have hardly changed their views even after 2 years, in spite of following the Bayesian logic. In contrast, if the prior probability for the incorrect choice *X* = 0 is reduced to, say, 80%, then with the same amount of information-revelation rate (σ = 1 in both cases) there will be more variabilities, as shown on the right panel.

The mechanism behind the tenacious Bayesian phenomenon can be explained by means of communication theory. It has been remarked that for the uncertainty to reduce on average to a fraction of the initial uncertainty, a typical timescale required for gathering information is proportional to the inverse square of the information flow rate σ. More precisely, the timescale is given by (σΔ)^−2^, where Δ^2^ is the initial uncertainty, measured by the variance. Hence if the prior probability is highly concentrated at one of the alternatives, then Δ is very small, so typically it will take a very long time for the initial uncertainty to reduce by a significant amount. This is not an issue if the initial inference is the correct one. However, if the initial inference is incorrect, then there is a problem, for, the uncertainty will have to significantly increase before it can decrease again. As a consequence, having a very high prior weight on any one of the alternatives means it is difficult to escape from that choice even if ultimately it is not the correct one, because each alternative acts like an attractor. Sample paths illustrating this effect are shown in Figure 3.

In the characterization of human behavior it is sometimes argued that people act in an irrational manner if they do not follow the Bayesian rule. So for instance if a person is presented with a fact that diametrically contradicts their initial view, and if the person does not change their view afterwards, then this is deemed counter to Bayesian and hence irrational. While it is not unreasonable to associate irrationality with a lack of Bayesian thinking, any experimental “verification” of irrational behavior based on this criterion is questionable, due to the tenacious Bayesian phenomenon. A good example can be seen in the aftermath of the 2020 US presidential election. Many believed (and still do) that the election outcomes were “rigged” even though the large number of lawsuits put forward challenging the outcomes were thrown out of courts one after another. Although the factual evidences presented suggested that the election results were not rigged, this had little influence on those who believed the contrary. One might be tempted to argue that this behavior is irrational, but a better characterization seems to be that these people are acting rationally in accordance wth their Bayesian logic, albeit they have strongly skewed priors.

It should be evident that the effect of fake news naturally is to exacerbate the issue associated with the concentration of prior weights on incorrect inferences. In particular, if the prior weight for an incorrect inference is already high, then it does not require a huge amount of disinformation to maintain this status. Therefore, the phenomenon of tenacious Bayesian behavior will have to be taken into account in exploring measures to counter the impacts of fake news.

One immediate consequence of the tenacious Bayesian behavior is that it explains, at least in part, the confirmation bias within the Bayesian logic. Broadly speaking, confirmation (or confirmatory) bias refers to a well-documented behavior whereby people with particular views on a given subject tend to interpret noisy information as confirming their own views (Klayman, 1995; Nickerson, 1998; Martindale, 2005). Thus, two people with opposing views, when presented with the same ambiguous information, may simultaneously interpret the information as supporting their initial views. If, in particular, the polarization of the two opposing views increases after digesting the same noisy information (Lord et al., 1979), then this is considered as a clear evidence that people do not follow Bayesian thinking (Griffin and Tversky, 1992; Rabin and Schrag, 1999; Dave and Wolfe, 2003; Nishi and Masuda, 2013; Rollwage and Fleming, 2021).

The tenacious Bayesian behavior observed here, however, suggests that such a phenomenon is not necessarily incompatible with the Bayesian logic, and hence that, contrary to common assertion, to a degree, confirmation bias can be explained as a consequence of Bayesian thinking. To establish that the tenacious Bayesian behavior is a generic feature of Bayesian updating under uncertainties, it is necessary to work directly within the state space of decision making, which will be explained now.

Suppose that the views held by decision maker *A* on a set of *n* alternatives is represented by the probabilities (*p*_1_, *p*_2_, …, *p*_*n*_), while that of decision maker *B* is represented by (*q*_1_, *q*_2_, …, *q*_*n*_). To determine the level of affinity it will be useful to consider instead the square-root probabilities $\psi_{i}=\sqrt{p_{i}}$ and $\phi_{i}=\sqrt{q_{i}}$. These square-root probabilities then represent the *states of decision makers*. More specifically, the state space of decision making is a vector space of unit-normalized positive vectors endowed with the Euclidean inner product, such that squared components of the vector determine probabilities for different alternatives. The separation of the two decision makers *A* and *B* can then be measured in terms of the spherical distance


$$\theta =\cos^{-1}\left(\overset{n}{\underset{i=1}{\sum }}\psi_{i}\phi_{i}\right),$$

known in statistics as the Bhattacharyya distance (Brody and Hook, 2009). A definite state of a decision maker is represented by elements of the form *e*_*k*_ = (0, …, 0, 1, 0, …, 0), where only the *k*-th element in *e*_*k*_ is nonzero. If two decision makers have identical views, then their separation distance vanishes, while if the distance takes its maximum value θ = π/2 then their views are orthogonal, and hence incompatible.

If the vector {ψ_*i*_} represents the prior state of decision maker *A*, and if noisy information relevant to the choice is revealed, then the prior will be updated to a posterior state {ϕ_*i*_} in accordance with the Bayes formula, in the sense that the transformation $\psi_{i}^{2}\to \phi_{i}^{2}$ is determined by the Bayes formula. Now in the continuous-time setup where the noise is modeled by a Brownian motion, it is known in signal detection that the transformation of the posterior probability is governed by a differential equation known as the Kushner equation (Kushner, 1964). Translating this equation into the state-space by use of the square-root map, one finds that the deterministic component (the drift) of the dynamics is given by the negative gradient of the variance of the signal that is to be inferred from the noisy information (cf. Brody and Hughston, 2002). The nature of a negative gradient flow is to push the state into another state of a lower variance. What this means is that if the state of a decision maker is close to one of the definite states, say, *e*_*k*_, for which the variance is zero, then the flow generated by Bayesian updating has the tendency of driving the state toward *e*_*k*_. Putting the matter differently, the definite states {*e*_*i*_} are the attractors of the Bayesian flow.

Now the variance is a measure of uncertainty, so this feature of Bayesian flow is only natural: reduction of uncertainty is what learning is about, and this is the reason why Bayesian logic is implemented in many of the machine learning algorithms, since the Bayesian updating leads to maximum reduction in uncertainty. However, this attractive feature can also generate an obstacle in the context of decision making, because the prior view held by a decision maker is subjective and hence may deviate far away from objective reality. In particular, if the state of a decision maker is close to one of the false realities *e*_*k*_, then the Bayesian flow will make it harder to escape from the false perception, although by waiting long enough, eventually a decision maker will succeed in escaping from a false attractor. Or, alternatively, if by a sheer luck the noise takes unusually large values that take the state away from the attractor, then by chance a quick escape becomes possible, but only with a small probability.

**Figure 4:**
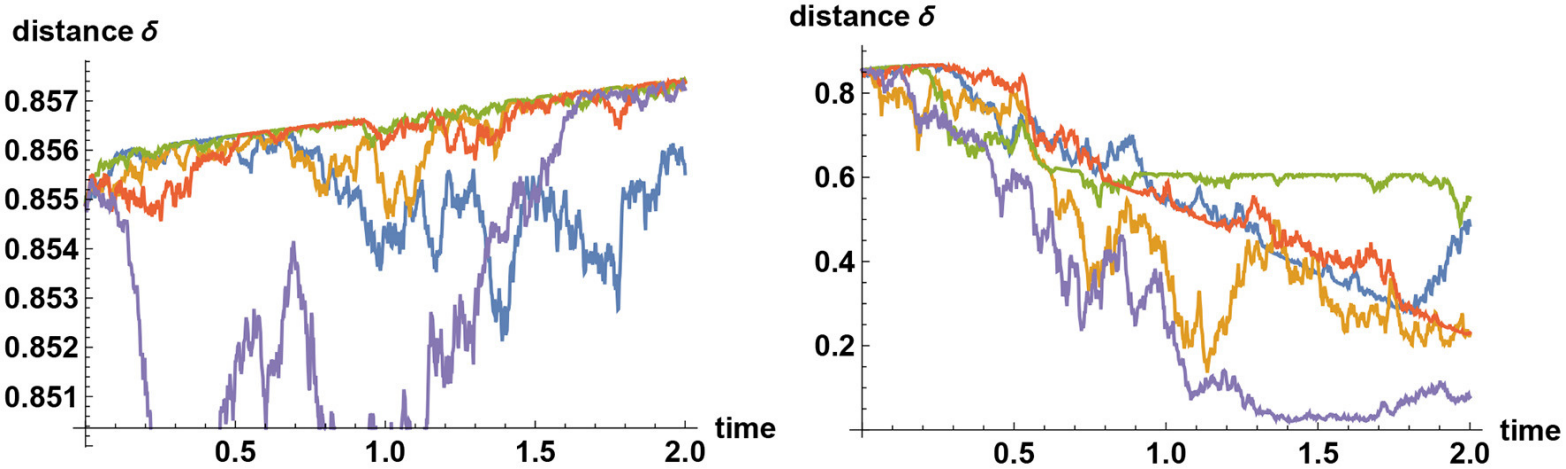
Separation distance under Bayesian updating. The polarity, or distance δ of two decision makers, when they are provided with an identical set of noisy information, has a tendency to increase under the Bayesian updating, even though on average it decreases in time. Five sample paths are shown here for two different choices of σ. On the left panel the information flow rate (signal to noise ratio) is taken to be σ = 0.2. Simulation studies (not shown here) indicate that in this case the upward trend persists for some 40 years in about 50% of the sample paths, and the separation distance is typically reduced to half of the initial value after about 100 years. When the information flow rate is increased eleven-fold to σ = 2.2, polarized Bayesian learners are forced to converge a lot quicker, as shown on the right panel, where the separation is reduced to half of its initial value typically within 2 years.

With these preliminaries, let us conduct a numerical experiment to examine how the separation of two decision makers evolve in time under the Bayesian logic. Specifically, let there be five possible choices represented by a random variable *X* taking the values (1, 2, 3, 4, 5). Decision maker *A* assigns 96% weight on the second alternative, whereas for other alternatives assigns 1% weight each. Similarly, decision maker *B* assigns 96% weight on the third alternative, whereas for other alternatives assigns 1% weight each. The initial separation of the two is thus given by θ≈1.343. Normalizing the separation by setting δ = 2θ/π so that 0 ≤ δ ≤ 1 we find that the initial separation distance is given by δ_0_≈0.855, where the subscript 0 denotes the initial condition. Both decision makers are provided with the same noisy information represented by the time series ξ_*t*_ = σ*Xt*+ϵ_*t*_, where the noise ϵ_*t*_ is modeled by a Brownian motion. The simulator can secretly preselect the “correct” decision to be, say, the fourth alternative so that both decision makers are trapped at wrong inferences. (The choice of the correct alternative will have little impact on the dynamics of the separation distance.) The results of numerical experiments are shown in Figure 4. It should be stressed first that *on average* the separation measure {δ_*t*_} is a decreasing process, because Bayesian updating forces decision makers to learn. Yet, simulation study shows that there is a clear trend toward slowly increasing the separation measure over shorter time scales. That is, the separation tends to increase slightly, but when they decrease, the amount of decrease is more pronounced that on average it decreases.

An important conclusion to draw here is that the separation of two decision makers *can* increase under Bayesian inferences. Thus, while there is no suggestion here that confirmation bias can be fully explained by means of Bayesian logic, the analysis presented here, based on the tenacious Bayesian phenomenon, shows that the gap between empirical behaviors of people and those predicted by Bayesian logic in the context of confirmation bias is significantly smaller than what is often assumed in the literature.

## 7. Psychology of False Belief

It is of interest to remark that methods of communication theory goes sufficiently far to allow for the simulation of an “alternative fact,” that is, the simulation of an event whose probability of occurrence, or the *a priori* probability perceived by the decision maker, is zero. In this connection it is worthwhile revisiting the meaning of the *a priori* probabilities. In the context of natural science, these probabilities are interpreted to represent the objective probability of an event taking place. Thus, if an event with a very low *a priori* probability were to occur, then the interpretation is the obvious one, namely, that a very rare event has occurred. In the context of social science, however, these probabilities need not characterize in any sense an objective reality. Hence, if a decision maker were to assign, say, a very low *a priori* probability on one of the alternatives, then the interpretation here is that the probability merely reflects the subjective perception of that decision maker, while in reality the objective probability of that alternative being selected may remain high. In other words, a false belief does not represent a rare event.

In an extreme case, a decision maker may assign zero probability to an alternative which may nevertheless represent reality. This can be viewed as an extreme limit of the tenacious Bayesian behavior, except that, perhaps surprisingly, Bayesian logic here predicts that the psychology of a decision maker with a perfect false belief (that is, someone who assigns zero weight on an alternative that represents physical reality) exhibits an erratic indecisive behavior different from the tenacious Bayesian characteristics. Such a behavior is seen, however, only when there are more than two alternatives, for, if there are only two alternatives and if the *a priori* probability is zero for one of them, then the view of the decision maker will not change in time under the Bayesian logic.

**Figure 5:**
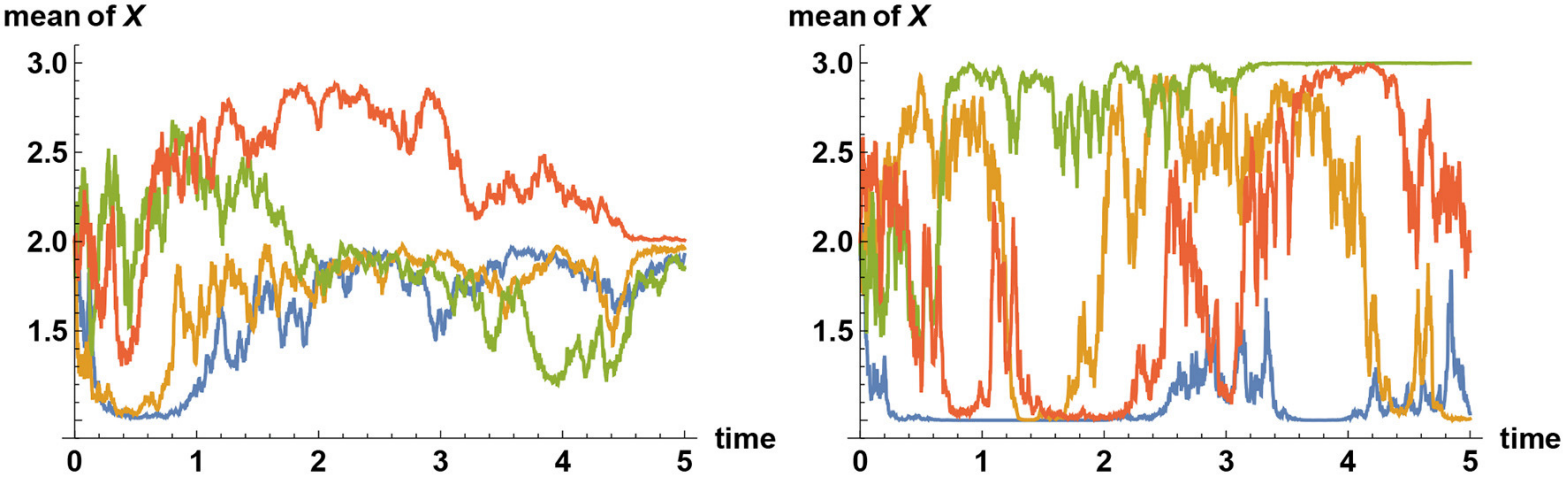
Simulating alternative fact. Three alternatives are represented by the values *X* = 1, *X* = 2, and *X* = 3. Plotted here are sample paths for the mean values of *X* subject to information process ξ_*t*_ = σ*Xt*+ϵ_*t*_, where σ = 2 and {ϵ_*t*_} denotes Brownian noise. In all simulations, the simulator has chosen the alternative *X* = 2 to be the correct one. On the left panel, all decision makers start with the prior belief that the probability of *X* = 2 is only 10%, whereas the two other alternatives are equally likely realized at 45% each. Hence the initial mean of *X* equals 2. Initially, their views tend to converge either to *X* = 1 or *X* = 3; but over time, sufficient facts are revealed that they all converge to the correct choice made by the simulator. But what if they do not believe in the “truth” at all? On the right panel, the simulator has again chosen *X* = 2 to represent the true value, but the decision makers assume that this is impossible and that the two other alternatives are equally likely realized at 50% each. Hence again the initial mean of *X* equals 2. In this case, the decision makers' views tend to converge quickly to one of the two “false” alternatives *X* = 1 or *X* = 3. These two beliefs are, however, only quasi-stable; the views will never converge indefinitely. Instead, their views tend to flip back and forth between the alternatives *X* = 1 and *X* = 3, but never converging to either one, and certainly never come close to the correct alternative *X* = 2.

Sample paths of such simulations are shown in Figure 5, in the case where there are three alternatives. In general, when there are more than two alternatives, and if decision makers assign zero prior probability to the true alternative, then their views tend to converge quickly to one of the false alternatives, remain there for a while, before it jumps to one of the other false alternatives and then after a while jumps again to yet another false alternative. Such a “hopping” phenomenon can only be observed when they categorically refuse to believe in or accept the truth, and this erratic behavior is predicted by Bayesian logic.

The intuitive reason behind this hopping behavior is that no false belief can ever be stable for long under the presence of information that reveals the reality. Hence the stronger is the information revelation rate about the reality, the more erratic the behavior becomes. This feature can be studied alternatively by examining the Shannon-Wiener entropy (Wiener, 1948), which represents the measure of uncertainty. Under a learning process characterized by the observation of the noisy time series {ξ_*t*_}, the uncertainty about different alternatives as characterized by entropy decreases on average. Hence a learning process is represented by the reduction of entropy, resulting in a low entropy state. This is why a decision maker who refuses to accept the real alternative quickly reaches a state of low entropy, and wishes to stay there. The reality however contradicts the chosen alternative. Yet, if entropy (hence uncertainty) were to now increase, even though the learning process continues, then this amounts to an admission of having to have rejected the truth. In other words, a state of high entropy is unstable in such a circumstance. The only way out of this dichotomy is to rapidly swap the chosen false alternative with another false alternative, until reality is forced upon the decision maker, at which point the second false alternative is discarded and replaced by either the original or yet another false alternative. This process will continue indefinitely. Only by a reinitialisation of the original assessment (for instance by a dramatic event that radically changes one's perception) in such a way that assigns a nonzero probability on the rejected alternative—no matter how small that may be—a decision maker can escape from this loop.

It might be worth pondering whether the assignment of strictly vanishing probability (as opposed to vanishingly small probability) to an alternative by a decision maker represents a realistic scenario. Indeed, it can be difficult to determine empirically whether a decision maker assigns strictly zero probability on an alternative, although in some cases people seem to express strong convictions in accepting or rejecting certain alternatives. Yet another possible application of the zero-probability assignment might be the case in which a decision maker, irrespective of their prior views, refuses to admit the real alternative. (For example, they have lied and then decide not to admit it.) Whether the behavior of such pathological liars under noisy unraveling of information about the truth can be modeled using the zero-probability assignment approach here is an interesting open question.

## 8. The Role of Noise

In Brody and Meier (2022) it is shown that even if a decision maker is unaware whether any given information is true or false, so long as they know the probability distribution of the fake news (represented by the time series {*f*_*t*_}), then this is sufficient to eliminate the overall majority of the impact of fake news. In other words, anticipation of fake news is already a powerful antidote to its effects. While this feature is encouraging, it can also act against defending the truth, for, politicians nowadays often quote the phrase “fake news” to characterize inconvenient truth statements. Hence for those who believe in unfounded conspiracies, they anticipate truths being revealed which they perceive as false, and this anticipation also acts as a powerful antidote against accepting reality. Is there an alternative way of tackling the issue associated with strongly polarized clusters then?

In this connection it is worth observing that the formation of domains and clusters described above is not uncommon in condensed matter physics of disordered systems. Here, atoms and molecules forming the matter interact with other atoms and molecules in their neighborhoods. An atom, say, will then attempt to take the configuration that minimizes the interaction energy with its neighbors (lower energy configurations are more stable in nature), but because this minimization is a local operation, inconsistencies will emerge at large scales, and clusters of locally energy-minimizing configurations will be formed. The boundary of different clusters, such as a domain wall, are called “defects” or “frustrations” in physics.

To attempt to remove a defect, one can heat the system and then slowly cool it again. What the thermal energy does is to create a lot of noise, reconfiguring atoms and molecules in a random way so that after cooling back to the original state, the defect may be removed with a certain probability. This is essentially the idea of a Metropolis algorithm in a Monte Carlo simulation. Hence although noise is generally undesirable, it can play an important role in assisting a positive change, albeit only with a certain probability.

There is an analogous situation that arises in biological processes (Trewavas, 2012, 2016). Most biological processes are concerned with either processing the information about the environments, or else copying genetic information. In either case, noise is highly undesirable under normal circumstances. However, when a biological system is faced with an existential threat, then the situation is different. By definition, in such a circumstance, the conventional choices made by a biological system that would have been the correct ones under normal conditions are problematic, and it may be that for survival, the system must make a choice that *a priori* seems incorrect. This is where noise can assist the system, to get over the threshold to reach unconventional choices. In other words, noise, as well as being a nuisance, is also what makes the system robust.

Returning the discussion to disinformation, it should be evident that the main issue is not so much in the circulation of “fake news” *per se*, but rather it is the coexistence of (a) polarized information clusters and (b) disinformation that creates real problems that are threats to democratic processes, or to public health. Hence to tackle the impact of disinformation a more effective way than the traditional “fact checking” strategy (which in itself of course has an essential role to play) seems to be in the dismantlement of the “defects” in the information universe, and this is where noise can potentially play an important role.

Of course, noise, having no bias, is unpredictable and the effect could have been the other way around. Nevertheless, without a substantial noise contribution the decision maker would have been stuck at a wrong place for a long time, and having a nonzero probability of an escape is clearly more desirable than no escape at all. In a similar vein, to dismantle an information cluster, rather than trying to throw factual information at it (which may not have an effect owing to the tenacious Bayesian phenomenon, and can also be costly), it may be more effective to significantly increase the noise level first, in such a way that decision makers are unaware of the increased level of noise, and then slowly removing it. The idea is to sufficiently confuse the misguided individuals, rather than forcing them to accept the facts from the outset. The result may be the resurgence of the original cluster, but there is a nonzero probability that the domain wall surrounding the cluster is dismantled. Putting it differently, an effective countermeasure against the negative impacts of disinformation might be the implementation of a real-life Metropolis algorithm or a simulated annealing (a slow cooling to reach a more stable configuration).

**Figure 6:**
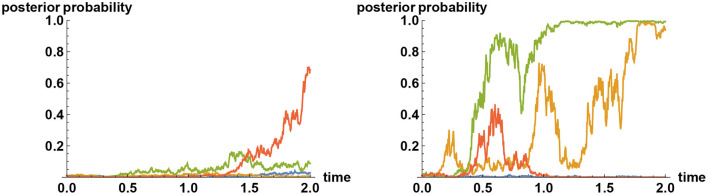
Tenacious Bayesian binary decision with enhanced noise. What happens to the tenacious Bayesian behavior of the left panel in Figure 3 if the noise level is enhanced in such a way that decision makers are unaware of it? As in the example of Figure 3, the decision makers here have their priors set at 99% for the choice *X* = 0, but the simulator has chosen *X* = 1 to be the correct choice. Plotted here are sample paths for the *a posteriori* probability that *X* = 1. In the left panel the noise level is doubled as compared to that of the left panel in Figure 3; whereas in the right panel it is quadrupled. In both cases, the information flow rates are the same as that chosen in Figure 3, so no more reliable information is provided here for the Bayesian decision makers. Nevertheless, the introduction of unknown noise enhances the chance of arriving at the correct decision considerably sooner, with positive probability. In particular, if the noise level is quadrupled, then there is about 15% chance that the noise will assist such an escape from a false reality after two years.

As an example, in Figure 6 the impact of noise enhancement, when the decision makers are unaware of the noise manipulation, is shown, when the noise level is doubled and when it is quadrupled. If the orientation of noise goes against the truth, then the noise manipulation merely enforces the tenacious Bayeian effect more strongly (like one of the sample paths in blue on the right panel), but there is an equal probability that noise goes the other way, in which case the views of decision makers are altered considerably.

## 9. Discussion: Possible Role of Utility

The theory of decision making under uncertainty is of course a well-established area of study in statistics (DeGroot, 1970; Berger, 1985). The theory outlined here departs from the traditional one by taking into consideration the flow of information that affects the perceptions of decision makers, thus allowing for an explicit dynamical characterization of decision makings. This, in turn, opens up the possibility of engaging in a comprehensive case studies and scenario analysis. In this context, it also becomes evident how information manipulation in the form either of the dissemination of disinformation or noise adjustment can be built into the modeling framework, because the starting point of the analysis is the specification of the flow of information.

Now in the context of statistical decision theory, the standard treatment presumes that an alternative is chosen if it maximizes the expected utility, or perhaps if it minimizes the expected loss (DeGroot, 1970; Berger, 1985). The utility function characterizes the preference profile of a decision maker. While rational choice as characterized by maximizing expected utility has been challenged (Kahneman and Tversky, 1979), the utility theory nevertheless works well in many applications. In particular, in the context of financial economics, correct valuations of assets are carried out by taking into account the impact of the utility. Putting the matter differently, when financial assets are priced by means of the expectation of the future return, this expectation is taken not with respect to the real-world probability, but rather, with respect to a risk-adjusted system of probabilities.

It may be that analogously, when analyzing, for instance, a voter's decision in an election it is more appropriate to consider a preference-adjusted probability associated with the utility profile of that voter, rather than the real-world probability. It is entirely possible, for instance, that some of the empirically observed phenomena such as confirmation bias can be explained even more accurately by combining the tenacious Bayesian behavior with utility optimisation. Should this be the case, however, the information-based approach outlined here remains applicable; one merely has to reinterpret the probabilities slightly differently, but the formalism itself remains intact, and so are the conclusions.

In summary, an information-based approach to characterizing the dynamics of systems driven by information revelation has been elaborated here in some detail using simple decision-making scenarios, and the impact of information manipulation, including dissemination of disinformation, and how such concepts can be modeled in a scientifically meaningful manner, has been clarified. The effect of having an excessively high weight placed on a false belief—called a tenacious Bayesian inference here—is explained, and an extreme case of the effect, what one might call an alternative fact, is simulated to uncover their erratic characteristics. In particular, it is shown, based on the tenacious Bayesian behavior, that confirmation bias can be explained, to an extent, within the Bayesian framework. Finally, a specific way of manipulating noise as a way of combatting the negative impact of disinformation is proposed.

The information-based approach developed here not only allows for a systematic study of the behaviors of people under uncertain flow of information, but also can be implemented in practical applications. For sure some of the model parameters such as σ and *f* need not be controllable globally, especially in the context of a competition whereby one has no control over the strategies of the competitors. Nevertheless, there are means to estimate model parameters. For instance, in the context of an electoral competition, by studying the variability (volatility) of the opinion poll dynamics, the information flow rate σ can be estimated quickly. Alternatively, model parameters may be calibrated from the response to changes in the strategy. For instance, in the context of marketing, one can ask how a 30% increase in advertisement cost influenced on the sales figure. From such an analysis one can infer the level of information flow rate. At any rate, the mere fact that the information-based approach makes it possible to conduct a comprehensive impact studies and scenario analysis in itself is a huge advantage in developing informational strategies.

## Data Availability Statement

The original contributions presented in the study are included in the article/supplementary material, further inquiries can be directed to the corresponding author.

## Author Contributions

The author confirms being the sole contributor of this work and has approved it for publication.

## Conflict of Interest

The author declares that the research was conducted in the absence of any commercial or financial relationships that could be construed as a potential conflict of interest.

## Publisher's Note

All claims expressed in this article are solely those of the authors and do not necessarily represent those of their affiliated organizations, or those of the publisher, the editors and the reviewers. Any product that may be evaluated in this article, or claim that may be made by its manufacturer, is not guaranteed or endorsed by the publisher.
